# Altering chromatic aberration: how this latest trend in intraocular-lens design affects visual quality in pseudophakic patients

**DOI:** 10.1186/s40662-023-00367-w

**Published:** 2023-12-12

**Authors:** Grzegorz Łabuz, Helin Güngör, Gerd U. Auffarth, Timur M. Yildirim, Ramin Khoramnia

**Affiliations:** The David J. Apple Center for Vision Research, Department of Ophthalmology, University Hospital Heidelberg, Im Neuenheimer Feld 400, 69120 Heidelberg, Germany

**Keywords:** IOLs, Chromatic aberration, Depth of focus, Contrast sensitivity

## Abstract

**Background:**

Chromatic aberration of the eye results from the dispersion of polychromatic light at the interfaces of ocular media. An intraocular lens (IOL) based approach utilizing the diffractive-multifocal principle has been proposed for its correction, but the clinical evidence on the impact of reducing or increasing chromatic aberration on the visual quality of pseudophakic patients remains scarce.

**Methods:**

In this cross-sectional study, longitudinal chromatic aberration (LCA) effects were studied monocularly in 37 patients implanted with a monofocal lens. LogMAR corrected distance visual acuity (VA) and defocus curve at the + 1.0 D to − 2.0 D range were assessed. Contrast sensitivity (CS) was evaluated at far and at four spatial frequencies. Measurements were performed with the eye's natural conditions, as well as with increased and corrected (by the same amount) LCA, which was altered by introducing zero-power trial triplets.

**Results:**

The mean (± standard deviation) logMAR VA was − 0.11 ± 0.07 for the natural condition, − 0.13 ± 0.07 for the LCA-corrected eye, and − 0.06 ± 0.08 for the eye with increased LCA. A sharp decline of the defocus tolerance was found after the LCA correction with the VA value of 0.38 ± 0.15 logMAR at − 1.5 D. However, for the natural and increased LCA, it was 0.32 ± 0.16 logMAR and 0.25 ± 0.13 logMAR, respectively. CS was improved at all spatial frequencies after the LCA correction, which was closely followed by the natural-eye performance. Increased LCA resulted in reduced CS, mainly at higher spatial frequencies.

**Conclusions:**

We demonstrated that elevating chromatic aberration above the natural level of monofocal patients extends their depth of focus while causing a measurable albeit minimal reduction in visual function. Still, the observed changes indicate that neither correction nor increase of LCA yields a substantial clinical effect on distance VA and CS in monofocal pseudophakia.

## Background

In general, chromatic aberration results from the dispersion of polychromatic light at the interfaces of refractive media. In the eye, as in other refractive systems, when polychromatic light passes through ocular media, the shorter wavelengths refract more strongly than longer wavelengths. The resulting shift between wavelengths causes a difference of focus, which is called longitudinal chromatic aberration (LCA), and also a difference in magnification, termed transversal chromatic aberration [1].

Transversal chromatic aberration depends primarily on the alignment of the pupil, the lens, and the cornea; thus, it is marked by higher inter-individual differences or even dissimilarity between the left and right eyes [1–3]. Despite experiencing a 1.7-fold increase during infancy [4], LCA shows only minimal variability in the adult population and remains largely unaffected by the aging process [5, 6]. In the human eye, an LCA of about 2 D has been found in the 400–700 nm range [7]. Despite this sizeable chromatic shift, the retinal spectral sensitivity mitigates the impact of LCA on polychromatic vision, which is comparable to a blurring effect of a spherical lens with < 0.25 D defocus [8]. Monochromatic aberrations may further reduce the LCA impact on the visual quality [9].

In pseudophakic eyes, the dispersive properties of an implanted intraocular lens (IOL) influence the eye's chromatism, and the IOL's Abbe number often quantifies the lens’ contribution. A high Abbe number indicates low material dispersion and vice versa. The reported LCA values for pseudophakic eyes range from 0.45 D to 1.45 D, which despite inter-subject variability, also depend on the lens model, the light spectrum, and the measurement procedure [10, 11]. The increasing awareness of the magnitude of LCA in the pseudophakic population has led to the current trend among IOL manufacturers to correct the IOL's and eye's chromatism in presbyopia-correcting IOLs through a diffractive principle [12–15]. Laboratory studies have shown that such designs indeed reduce the impact of LCA on the polychromatic image quality when tested in a model eye [12–15].

In recent years, IOLs that claim to correct the eye's chromatic aberration have become available to cataract and refractive surgeons. The primary rationale for using these IOLs is based on laboratory measurements of optical and visual quality [12, 15, 16]. Clinical studies have yet to show either clear benefits of this technology or an absence of adverse effects. There are limits to the clinicians' ability to test how the features of these new IOLs affect patient's vision. At present, one evaluates multifocal-IOL patients' visual performance by measuring visual acuity (VA), contrast sensitivity (CS), defocus curve, and aberrometry [17]. These are helpful measurements when comparing different IOLs, but finding the impact of chromatic aberration on vision may be confounded by the inter-subject variability or the presence of lower- and higher-order monochromatic aberrations [9–11, 16]. It would be desirable to find a procedure where we could measure the LCA's impact as perceived by a patient; thus, including the patient's chromatic and monochromatic aberrations as well as the contribution from adaptation mechanisms. Ideally, such a method will involve using lenses with varying chromatic aberration levels fitted into a trial frame.

Here, we followed a standard clinical protocol for premium IOL assessment, and we used two trial lenses (one which reduces and another that increases the eye's LCA) to address the following questions: (1) Does chromatic aberration significantly affect visual function in patients with monofocal IOLs? (2) How does chromatic aberration affect the depth of focus in monofocal patients?

## Methods

Patients were recruited at the International Vision Correction Research Center of the University Eye Clinic Heidelberg. The study procedures adhered to the tenets of the Declaration of Helsinki and were conducted under Ethical Committee's approval (S-392/2011) granted by Heidelberg Medical Faculty's review board. Informed consent was obtained from all participants. The study cohort comprised of patients who had undergone routine cataract surgery and received a monofocal IOL. Only patients with a good VA of 0.16 logMAR or better were recruited. We excluded cases with ocular comorbidities, such as age-related macular degeneration (AMD), diabetic retinopathy, or glaucoma. We included only patients with a hydrophobic IOL since, in an earlier study, we had found a higher LCA level in a hydrophobic acrylic material compared to a hydrophilic one, on average, by 0.41 D [12, 18]. Still, in that work, the hydrophobic IOLs demonstrated a significant variation as a result of the diversity in their Abbe numbers, which was an additional factor taken into consideration when choosing the IOLs for the current study.

Thirty-seven patients with the following IOL models were included:

- Clareon CNA0T0 (Alcon Inc., USA).
- Vivinex XY1 (HOYA, Japan).
- Avansee CP2.2R (Kowa Co. Ltd., Japan).

All IOLs are made of hydrophobic-acrylic material and are deemed glistening-free [19–21]. The Clareon and the Vivinex IOLs share the same refractive index of 1.55 and have a comparable Abbe number of 36.3 ± 0.7 [22] and 36.9, respectively. The Avansee's refractive index is 1.52, which results in a slightly higher Abbe number, i.e., 42.

### Chromatic aberration modification

In advance of using the trial lenses with patients, we first designed zero-power achromatizing and chromatizing triplets to alter the eye's chromatic aberration. A virtual eye was built using OpticStudio (Radiant Zemax LLC, USA) based on an anatomical model described by Liou and Brennan with chromatic dispersion data from Atchison and Smith [23, 24]. An IOL with + 20.0 D nominal power and the dispersion properties of the Clareon material was used to simulate pseudophakia. The chromatic aberration of the model eye was assessed using a chromatic-focal shift tool built in OpticStudio within a 486 nm to 656 nm range.

**Fig. 1:**
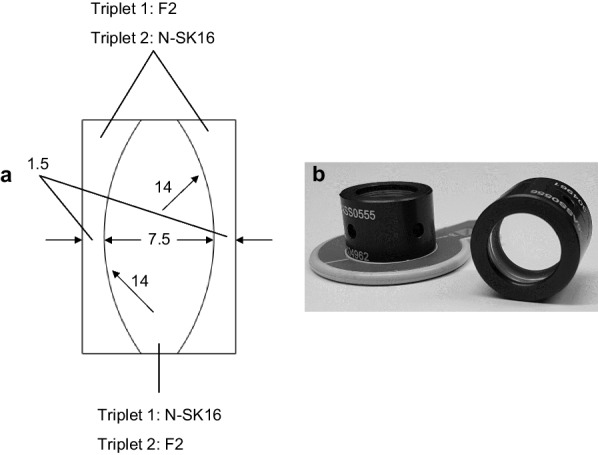
Design and fabrication of lenses to alter longitudinal chromatic aberration in vivo. **a** Schematic drawing with material characteristics of zero-power lenses designed to correct (Triplet 1) and increase (Triplet 2) chromatic aberration. All numerical values are given in millimeters. **b** Photographs of manufactured lenses fitted into a trial-lens mount

To change the model eye's chromatic aberration, we used a triplet lens with no optical power; thus, it could not alter the eye's refraction. This concept was first described by Bedford and Wyszecki [7], which was modified to fit into the then currently available optical glass materials. Although this lens was introduced to reduce the natural eye's chromatic effects, the increase of chromatic aberration is also possible by swapping lens material between the center and outer optical elements. In this configuration, a triplet lens induced approx. 1.00 D of chromatic aberration in the model eye. Figure 1 shows the design parameters of the zero-power triplets. The materials used for the cemented elements share a 1.62 refractive index but differ in chromatic dispersion with an Abbe number of 36.4 and 60.3 for F2 and N-SK16 glass, respectively.

On completion of the triplet testing in the virtual eye, the lenses were produced by an optical workshop (Sill Optics GmbH & Co. KG, Germany). The triplet was placed in a customized holder that fits into a standard trial frame used for routine refraction measurements.

One limitation of the Bedford and Wyszecki lens is that it is affected by transverse chromatic aberrations, which results in the appearance of fringes of color beyond about 1° of the visual field [7, 25–28]. Although these optical effects were later corrected by introducing an additional air-spaced (14.5 mm) doublet by Powell [26], the implementation of this approach would significantly increase the length of our optical system, making it unfeasible to be used with the trial frame. Given the primary objective of integrating the triplets in the routine clinical examination and that the angular range of VA and CS measurements falls < 1° field size, we decided not to apply Powell's modification in our system.

### Study protocol

The clinical evaluation was split into two parts. First, we tested the ability of the triplet lenses to correct and increase LCA. Then, the impact of chromatic effects on visual function in patients with monofocal IOLs was studied. Given the lengthy and extensive nature of each visual examination, we required no patient to participate in both parts of the project.

After confirming patients' eligibility, ten patients were selected for the assessment of LCA in both eyes. We determined the eye's chromatic shift by measuring the patient's refraction at two spectral conditions. First, best-corrected VA (BCVA) was measured with an Early Treatment Diabetic Retinopathy Study (ETDRS) chart placed at 4 m. Chromatic aberration (natural viewing) was determined at the patient's far point following the procedure described by Campbell et al. [29]. To this end, we used two spectral filters: one was a short pass (blue) filter with a 500-nm cut-off wavelength (FES0500, Thorlabs, USA), and the other was a red glass used for a duochrome test, which was a long pass filter with a 605-nm cut-on wavelength, as shown by our spectral measurements (Humphrey Optical Lens Analyzer, Carl Zeiss Meditec, Germany). The patients’ final spectacle correction was adjusted with a ± 0.125 D trial lens to improve the tests’ accuracy.

Once we determined the patient’s natural chromatic aberration level, a zero-power lens with positive or negative dispersion was introduced. We took special care to center the triplet lenses precisely to ensure that no color fringes overlay the test optotypes [25, 27, 28, 30]. Also, patients were informed about peripheral color fringes and instructed to disregard those optical effects. We asked them to move their heads rather than their eyes to change their fixation point during testing [25, 27, 28, 30]. Then, the same procedure as for the natural viewing was applied. Thus, in this part, three chromatic aberration values were obtained for (1) natural viewing, (2) the zero-power lens with increased and (3) reduced chromatic aberration.

We evaluated 32 patients to see how LCA affects patients’ visual quality and the depth of focus. However, we excluded five patients due to patient dropout (n = 3) or response bias (n = 2) during the study. For both eyes, we determined logMAR BCVA and then chose the eye with the better BCVA for further examination. Then, we obtained the defocus curve under three measurement conditions: natural viewing, increased and decreased chromatic aberration. The order of the three conditions was randomized using dedicated software.

We obtained a monocular defocus curve in patients wearing their best spectacle correction. The examination started at + 1.0 D and proceeded to − 2.0 D with a 0.5 D increment using trial lenses. The optotypes were randomized using a Clinical Trial Suite (M&S Technologies, USA), which is a computerized visual-testing platform. The device prevents patients’ memorizing bias by randomizing the display of high-contrast letters. Following successful measurements of the three defocus curves, CS was assessed under natural viewing, increased, and corrected chromatic aberration with a CSV-1000E (VectorVision Co, USA). The device consists of sinewave grating to assess the contrast threshold at 3, 6, 12, and 18 cyc/deg under photopic conditions (85 cd/m^2^ background luminance) without a glare source. Given the extensive nature of the evaluation, the CS dataset contains a lower number of patients due to missing or unreliable results. Photopic and scotopic pupil diameters were measured using an infrared pupilometer (NeurOptics, USA).

### Statistical analysis

Data analysis and visualization were performed with MATLAB (Mathworks, Inc., USA). We followed the method for the area under the defocus curve (AUDC) calculation described by Buckhurst et al. [31]. However, instead of 9th-order polynomial fitting to the defocus curves, we applied a Smoothing Splines algorithm implemented in MATLAB, which, based on visual inspection of data fitting, proved more robust at discrete points. Nevertheless, both approaches yielded excellent goodness of fit with an average R-squared parameter of 0.998 (Smoothing Splines) and 0.985 (polynomial). The far and intermediate AUDCs were tested at + 0.5 D to − 0.5 D and − 0.5 D to − 2.0 D range, respectively. The cut-off VA for the AUDC calculation was 0.30 logMAR [31].

The CS comparison between the test conditions was performed based on the area under the log contrast sensitivity function (AULCSF) parameter [32], integrated over the 3rd-order polynomial fit. This parameter was computed over a log spatial frequency ranging from 0.48 to 1.26 cyc/deg.

The normal distribution of analyzed data was confirmed by the Kolmogorov–Smirnov test and inspection of the Q-Q plot. The numerical values were summarized with the mean (± standard deviation). The AUDC and AULCSF measured under different chromatic-aberration states were compared using a repeated-measures ANOVA since each patient served as its own control. The post-hoc analysis was performed with a Bonferroni correction. In this pilot study, the significance level was set at 5%.

## Results

In total, 37 patients were assessed with a mean age of 70.2 ± 9.3 years. Subjects' spherical equivalent was − 0.71 ± 1.26 D, and BCVA was − 0.07 ± 0.10 logMAR. The nominal power of the implanted IOL was 19.8 ± 3.5 D. Patients' photopic and scotopic pupil size was 3.1 ± 0.6 mm and 4.7 ± 1.0 mm, respectively.

Of the 37 patients, 10 were randomly selected for chromatic aberration assessment (Vivinex: n = 10, Clareon: n = 10). The LCA found in those pseudophakic eyes was 1.08 ± 0.25 D. A zero-power lens designed to increase LCA elevated its level to 2.07 ± 0.30 D. However, a mean value of 0.12 ± 0.15 D was obtained in those patients after correcting their eye chromatism.

**Fig. 2:**
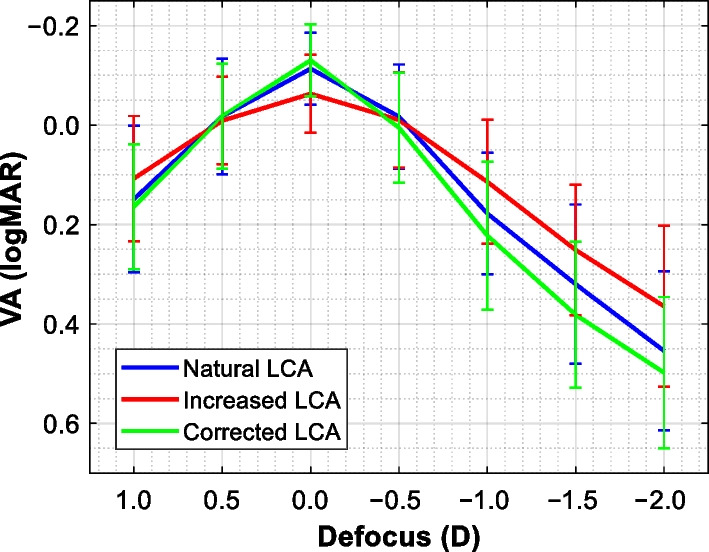
Defocus curves of patients measured with natural (blue), increased (red), and corrected (green) longitudinal chromatic aberration (LCA). Error bars = standard deviation; N = 27

VA and defocus curve were tested under the three chromatic conditions in the remaining 27 patients (Vivinex: n = 12, Clareon: n = 13, Avansee: n = 2). A BCVA of − 0.11 ± 0.07 logMAR was found in patients with their native chromatic aberration. One letter improvement was noted, on average, after correcting the eye's chromatism with − 0.13 ± 0.07 logMAR. It decreased by three letters with a nearly doubled LCA to a level of − 0.06 ± 0.08 logMAR. Figure 2 shows the average defocus curve for each condition. In this graph, notice a sharp decline of patients' VA following the correction of natural LCA with the mean value of 0.38 ± 0.15 logMAR at − 1.5 D. However, for natural viewing, 0.32 ± 0.16 logMAR was observed, on average. Increased LCA resulted in 0.25 ± 0.13 logMAR indicating half- and one-line improvement compared to natural and LCA-corrected viewing, respectively.

The distance AUDC for the natural and reduced-LCA condition was 0.38 ± 0.08, but for increased LCA it was 0.34 ± 0.08. Although these are minor differences, they were statistically significant. However, the post-hoc analysis revealed that statistical significance exists only between the measurements taken under increased LCA and natural (*P* = 0.002) or corrected LCA (*P* < 0.01) conditions. At the intermediate range, the AUDC obtained without zero-power lenses was 0.17 ± 0.10, which decreased to 0.13 ± 0.08 after LCA correction. However, the largest area of 0.22 ± 0.13 was found in those patients with a nearly two-fold increase of LCA. The difference between the three groups was statistically significant, which remained significant for the direct comparison between corrected LCA and the other two conditions (*P* ≤ 0.008), and it was also significant when the natural and increased-LCA AUDC were compared (*P* = 0.03).

**Fig. 3:**
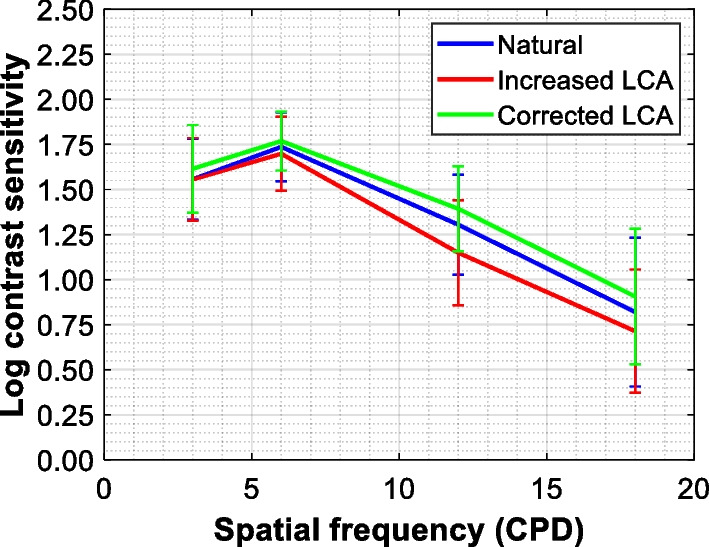
The impact of chromatic aberration on contrast sensitivity. The blue line indicates measurements performed under natural longitudinal chromatic aberration (LCA). The red and green lines refer to contrast sensitivity outcomes obtained following LCA's increase and decrease, respectively. Error bars = standard deviation; N = 20

Figure 3 shows the CS data assessed at four spatial frequencies in 20 eyes. The eye's chromatic correction yielded the highest CS, which was followed by the levels obtained under natural viewing. The lowest contrast-sensitivity values were found at all spatial frequencies but one (3 cyc/deg) after increasing the patients' chromatic aberration. The AULCSF analysis demonstrated that the observed differences are statistically significant, as the average values were 1.18 ± 0.17 for natural, 1.13 ± 0.16 for increased, and 1.23 ± 0.14 for corrected chromatic aberration. The post-hoc comparison confirmed a statistically significant contrast-sensitivity loss after LCA increased with respect to the natural (*P* = 0.001) and corrected-LCA (*P* < 0.001) levels. The mean difference between the latter two measurements was small but still significant, as the *P* value was 0.02.

## Discussion

We demonstrated that doubling LCA yields the depth of focus extension in monofocal-IOL patients. Although increased LCA resulted in a lower VA and CS, the patients' vision was still within the intersubject variability. In addition, the LCA correction significantly reduced the eye's intermediate range with scarce visual benefits at the far distance.

In 1957, Campbell performed a series of psychophysical experiments to determine parameters affecting the eye's depth of field [25]. The effect of LCA was tested by means of an achromatizing lens, which, as we observed in the current study, also demonstrated the reduction of the depth of field after the chromatic-aberration correction [25]. An analogous observation was made by Bobier et al. using a comparable methodology, which also showed that the depth-of-field might increase after LCA is increased from the natural level [30]. In a recent study, Suchkov et al. applied a phase modulator by modeling a diffractive element to alter subjects' LCA [33]. They found that the double amount of LCA may increase the depth of focus, with the LCA reduction yielding the opposite effect. However, none of those studies evaluated the impact of LCA in pseudophakic eyes. All testing was confined to a laboratory setting involving only a few (from one to four) phakic subjects, often individuals already experienced with psychophysical testing. In this study, we assessed 27 patients following a standard protocol for premium IOLs. We demonstrated that a two-fold increase of the LCA results in an apparent defocus-curve extension compared to the natural level, but the largest difference was observed between the achromatized eye and one with increased LCA. Thus, this study provides further evidence for the LCA potential to enhance the depth of focus and demonstrates how the visual range depends on the LCA level in pseudophakic eyes.

There have been attempts to improve the eye's VA through achromatization and of note is the work reported in 1967, where Campbell and Gubisch assessed the improvement of contrast thresholds under corrected and natural LCA and found the ratio of 1.04, concluding that the improvement was insignificant [29]. The comparison of the AULCS measured in the current study also yields a 1.04 ratio. Although we found this difference to be statistically significant, the expected impact on a patient's functional vision may indeed be negligible. A more substantial AULCS reduction was observed with the increase of LCA, but still, the CS values at each frequency were within the standard deviation of the natural CS measurements. For comparison,  the spectral dependency of a diffractive extended depth of focus (EDoF) IOL yields a VA loss of − 0.06 logMAR and − 0.09 logMAR at intermediate and near range, respectively, when longer (> 580 nm) than a designed wavelength is used [34]. A smaller effect was observed in patients following their LCA increase.

Bradley et al. [28] postulated several factors that might explain why there is a lack of significant visual improvement after achromatizing the eye. (1) A fixed correction cannot fully compensate for LCA, which results in a residual value of 0.00 to 0.20 D [27]. Here, it was 0.12 ± 0.15 D indicating a slight under-correction of the eye's chromatism. (2) Chromatic dispersion's impact is insignificant [1]; thus, either the correction or increase of LCA may only slightly affect the visual quality, which agrees with our results. (3) Achromatizing lenses do not correct but rather (4) induce transverse chromatic aberration, which, however, can be minimized if the lens is positioned close to the eye, correctly centered, and the test subtends less than 1° of the visual angle [25, 26, 30]. Although the simultaneous correction of monochromatic and chromatic aberrations may further improve VA and CS [16], making a clinical application of this approach is not feasible. Given, however, a minimal effect of LCA on the visual quality, also confirmed in a recent simulation study [8], increasing its level in monofocal patients rather than reducing it might have the advantage of extending the eye's intermediate range. The EDoF effect can be particularly appreciated when the corrected and increased LCA conditions are directly compared with at least one-line VA improvement at the defocus ≥ 1.0 D.

The AUDC, first described by Buckhurst et al., confines the description of a standard defocus curve to a single metric [31], which facilitates the statistical analysis and the comparison between the various lens models. In their monofocal group, Buckhurst and colleagues found the far and intermediate AUDC of about 0.32 ± 0.04 and 0.18 ± 0.04, respectively (refer to Fig. 3 of their study) [31]. For the natural condition, we found 0.38 ± 0.08 at far and 0.17 ± 0.10 at intermediate, which are close to the levels reported by Buckhurst et al. in their binocular assessment. Given that our measurements were performed monocularly, the observed AUDC improvement under doubled LCA could, in binocular vision, potentially increase by a factor of 1.07 due to binocular summation [35].

A new concept of monofocal IOLs with enhanced intermediate range, called 'monofocal plus', has been introduced in recent years [36, 37]. Those lenses feature a higher-order aspheric surface to extend the depth of focus and, at the same time, maintain a monofocal-lens optical quality [36, 37]. The focus extension in such lenses is, however, below a range of standard EDoF IOLs [38], and this has necessitated the creation of a new IOL classification. In a European multicenter study, Auffarth et al. compared a standard monofocal lens against one with an enhanced intermediate range in their clinical evaluation after cataract surgery [36]. They reported an average value of − 0.06 ± 0.01 logMAR after implanting the standard lens – the level identical to that found in our patient group with increased LCA. In a monofocal-plus group, the corrected distance VA was − 0.02 ± 0.01 logMAR, indicating a mere loss of 0.04 logMAR [36], similar to a difference observed in VA measured under natural and increased LCA (i.e., 0.05 logMAR). At 1.5 D of defocus, Auffarth and co-investigators found 0.31 ± 0.02 logMAR for the standard monofocal IOL [36], close to the level found in the current study under natural conditions, and 0.19 ± 0.02 logMAR for the enhanced monofocal lens. Thus, implementing a higher-order aspheric approach yields a larger focus extension than a doubled amount of natural LCA. However, whether the further increase of LCA could match the EDoF effect of a monofocal-plus lens will have to be addressed in future studies.

Many researchers have assessed the chromatic-aberration effects of modern IOLs [10–12]. Siedlecki et al. measured pseudophakic LCA using a visual refractometer and narrow-band diodes with a central wavelength of 470 nm, 525 nm, and 660 nm [10]. Chromatic difference of refraction between the blue and red condition was 1.45 ± 0.42 D and 1.17 ± 0.52 D for two IOL models made of AcrySof material (Alcon Inc., USA) which has similar dispersion characteristics to the Clareon model that we used. In a subsequent study, Nakajima et al. applied a Hartmann-Shack wavefront apparatus to determine LCA in subjects between 561 and 840 nm [11]. Following the conversion of their results to the spectral range of the Siedlecki et al. study, they reported 1.30 ± 0.40 D in their patients implanted with AcrySof IOLs and 1.16 ± 0.37 D with the Vivinex. However, their measurements of those IOLs in an artificial eye model showed an LCA of 0.97 D for both IOL models with a + 20.00 D nominal power and the 561–840 nm range, indicating the possible variability of the eye's chromatic aberration in the studied population [11]. We also evaluated the chromatic dispersion's effect on the patient's refraction in blue and red light; however, it was performed only to estimate the performance of the two triplet lenses with respect to the natural condition. We did not intend to evaluate the LCA in our patients precisely, as it was not the subject of the current study. Although the obtained LCA of 1.08 ± 0.25 D falls within the range reported by Siedlecki et al. and Nakajima et al., the wavelength at which it was determined cannot be accurately determined due to the use of the short- and long-pass filters. With the edge-pass filters, we could make the assessment without additional compensation for the sensitivity loss at both ends of the visible spectrum, and thus we could use standard clinical equipment. Hence, despite these similarities, we consider that our results should be used only to gauge the effectiveness of the triplet lens in the reduction or elevation of the eye's chromatic difference of focus.

Another limitation was the lack of information on the distribution of higher-order aberrations in our population, given the interplay between monochromatic and chromatic aberrations. Assessing this relationship could provide additional insight into the clinical significance of differences in chromatic-aberration effects depending on monochromatic aberrations and merits further investigation. Moreover, incorporating LCA corrections tailored to each participant's LCA level may provide a more robust validation of the study results, which warrants further research.

## Conclusions

Pseudophakic eye's chromatism could be increased or reduced with dedicated trial lenses that can be integrated into the procedures for standard clinical testing. Doubling the LCA in monofocal patients resulted in a depth-of-focus extension and within-the-norm outcomes for VA and CS, albeit with a measurable reduction. Although those visual-quality metrics improved following the eye's achromatization, the observed difference might not provide an appreciable effect for a patient. On the other hand, it yielded an apparent loss of defocus tolerance, which is already scarce after monofocal-IOL implantation. Whether a monofocal IOL with tripled or quadrupled LCA could further extend patients' visual range remains an open question. How non-native LCA levels might affect the perception of photic phenomena also requires the close attention of manufacturers planning to fully develop such an IOL. Another critical question is how the LCA affects vision in patients with a bifocal or trifocal IOL, given the broader defocus range and a narrower light-energy distribution of these lenses which will be the subject of subsequent work.

## Data Availability

The datasets used and analyzed for the present study are available from the corresponding author upon reasonable request.

## Abbreviations

CS: Contrast sensitivity
IOL: Intraocular lens
LCA: Longitudinal chromatic aberration
VA: Visual acuity
BCVA: Best-corrected visual acuity
AUDC: Area under the defocus curve
AULCSF: Area under the log contrast sensitivity function
